# From Hypothalamic Obesity to Metabolic Dysfunction-Associated Steatotic Liver Disease: Physiology Meets the Clinics via Metabolomics

**DOI:** 10.3390/metabo14080408

**Published:** 2024-07-26

**Authors:** Amedeo Lonardo, Ralf Weiskirchen

**Affiliations:** 1 Department of Internal Medicine, Azienda Ospedaliero-Universitaria of Modena (-2023), 41126 Modena, Italy; 2 Institute of Molecular Pathobiochemistry, Experimental Gene Therapy and Clinical Chemistry (IFMPEGKC), Rheinisch-Westfälische Technische Hochschule (RWTH), University Hospital Aachen, D-52074 Aachen, Germany; rweiskirchen@ukaachen.de

**Keywords:** craniopharyngioma, cirrhosis, fatty liver, history of medicine, hypopituitarism, hypothalamic syndrome, lipotoxicity, metabolomics, precision medicine

## Abstract

Metabolic health is tightly regulated by neuro-hormonal control, and systemic metabolic dysfunction may arise from altered function of the hypothalamic–anterior pituitary axis (HAPA). Ancient experimental observations of hypothalamic obesity (HO) and liver cirrhosis occurring among animals subjected to hypothalamic injury can now be explained using the more recent concepts of lipotoxicity and metabolic dysfunction-associated steatotic liver disease (MASLD). Lipotoxicity, the range of abnormalities resulting from the harmful effects of fatty acids accumulated in organs outside of adipose tissue, is the common pathogenic factor underlying closely related conditions like hypothalamic syndrome, HO, and MASLD. The hormonal deficits and the array of metabolic and metabolomic disturbances that occur in cases of HO are discussed, along with the cellular and molecular mechanisms that lead, within the MASLD spectrum, from uncomplicated steatotic liver disease to steatohepatitis and cirrhosis. Emphasis is placed on knowledge gaps and how they can be addressed through novel studies. Future investigations should adopt precision medicine approaches by precisely defining the hormonal imbalances and metabolic dysfunctions involved in each individual patient with HO, thus paving the way for tailored management of MASLD that develops in the context of altered HAPA.

## 1. Introduction

Metabolic health is tightly regulated by neuro-hormonal control. Therefore, impaired functional activity of the hypothalamic–anterior pituitary axis (HAPA) may result in systemic metabolic dysfunction and the deposition of ectopic lipids along hormonal routes [1]. It is logical to assume that dysregulated HAPA is causally involved in a fraction of cases of metabolic dysfunction-associated steatotic liver disease (MASLD), which is a lipotoxic disease [2]. Research conducted in the first half of the 20th century, reporting unexplained liver outcomes among animals subjected to induced thalamic injury, strongly supports this notion [3].

This perspective article aims to relate and reconcile historical experiments with recent pathomechanisms and nosography entities, i.e., lipotoxicity and MASLD, respectively. Here, we address forms of MASLD associated with hypothalamic obesity (HO) and MASLD.

## 2. Historical Background

In 1944, Brobeck reported that HO occurred because of rapid, transient, and intense hyperphagia, locomotory inactivity, and temporary depression of basal heat production in monkeys, dogs, cats, and rats subjected to experimental hypothalamic injury. Brobeck also discovered that HO was associated with “changes in the intermediary metabolism of carbohydrate which are the result of habitual overfeeding…. yellow liver… double the normal size… with the total quantity of hepatic fat … approximately four times the normal amount. Plasma lipid concentrations are likewise elevated.” [3]. This researcher pinpointed that the experimental findings in these mammals could safely be extrapolated to the human syndrome of HO, first described in 1840 by Mohr in patients with hypothalamic or pituitary disorders [3]. However, the significance of liver cirrhosis found in obese dogs with combined hypophyseal and hypothalamic injury was uncertain at that time, possibly attributable, according to Brobeck, to “a relative deficiency of lipotropic substances in the diet…. and worthy of further investigation” [3].

In 1994, Lee et al. proposed that a “lipotoxic” cause could underlie the dysfunction of pancreatic β-cells, explaining key steps in the pathogenesis of type 2 diabetes (T2D) [4]. Lipotoxicity defines the detrimental effects of fatty acids accumulated in various ectopic, non-adipose organs. These fatty acids damage the functions and anatomy of the pancreatic islets, heart, liver, kidneys, perivascular tissue, and skeletal muscle, accounting for the clinical manifestations of metabolic syndrome [5,6].

In 2004, Adams and colleagues conducted a groundbreaking study describing 21 patients (aged 3–66) who developed MASLD a median of 3 years after being diagnosed with hypothalamic and pituitary dysfunction. This condition was characterized by rapidly occurring obesity, insulin resistance, impaired glucose tolerance, and atherogenic dyslipidemia [7]. The authors ruled out other potential explanations for MASLD development, such as corticosteroid replacement therapy, and emphasized that their case study demonstrated an association, not causality, between neuroendocrine dysfunction and MASLD [7]. Subsequent investigations in 2006 and 2009, including those by Lonardo and Loria, contributed to the concept of a “hormonocentric” pathogenesis of MASLD and metabolic dysfunction-associated steatohepatitis (MASH) [8,9].

In 2009, Lee et al. illustrated HO as a condition burdened with significant morbidity and mortality, traditionally refractory to dietary and lifestyle changes, occurring in the context of hyperphagia, dysautonomia, reduced energy expenditures, hormonal deficits, and insulin resistance. These factors result from the combined contributions of dysregulated control of satiety, hunger, and energy balance, along with hormonal deficits due to hypopituitarism [10].

Collectively, these pioneering findings and novel paradigms have paved the way for improving our current understanding of hypothalamic syndrome and the etiology of HO (Table 1).

**Figure 1:**
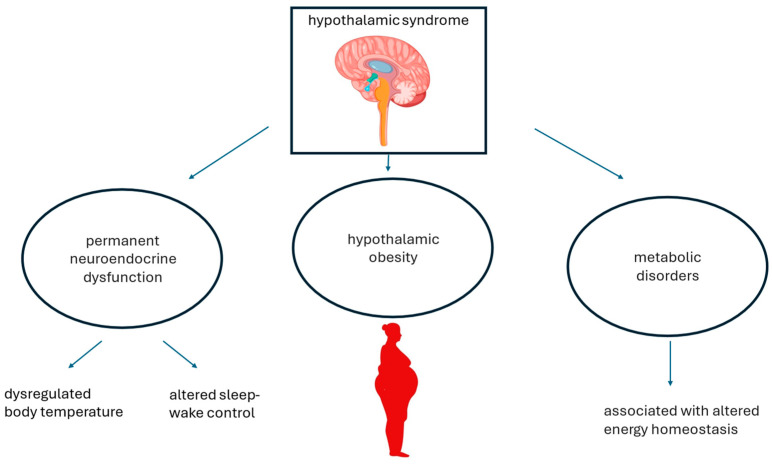
Hypothalamic syndrome. Hypothalamic syndrome is caused by various factors, as listed in Table 1, which ultimately result in neuroendocrine dysfunction, HO, and secondary metabolic disorders.

Hypothalamic syndrome, which may arise because of direct hypothalamic damage from cancer or as iatrogenic complications of neurosurgery and radiotherapy, typically involves a triad of neuroendocrine dysfunction HO and secondary metabolic disorders (Figure 1) [13].

In turn, HO is a rare and distinct type of obesity characterized by disrupted cerebral regulation of energy balance, autonomic nervous system function, and peripheral hormonal signaling [11].

## 3. Metabolic Dysfunction-Associated Steatotic Liver Disease

A prototypic example of lipotoxic disease, MASLD is closely and bidirectionally associated with diabetes, obesity, and other features within the realm of metabolic syndrome [2,14]. MASLD supersedes NAFLD by considering the presence of cardiometabolic risk factors and is categorized into various disease subgroups, including MASLD due to specific etiology [15].

The cellular and molecular mechanisms of lipotoxicity in MASLD include increased circulating concentrations of free fatty acids (FFAs). These FFAs induce hepatocyte apoptosis by activating the proapoptotic protein Bax in a c-jun N-terminal kinase-dependent manner. Furthermore, FFAs trigger the lysosomal pathway of cell death, regulate the expression of death receptor genes, promote endoplasmic reticulum stress, and trigger oxidative stress [16]. These pathomechanisms, along with others originating in the mitochondria, adipose tissue, and gut, converge to determine the development of MASLD and its progression to MASH [17].

## 4. Putative Pathomechanisms Associating Hypothalamic Obesity and Metabolic Dysfunction-Associated Steatotic Liver Disease

**Figure 2:**
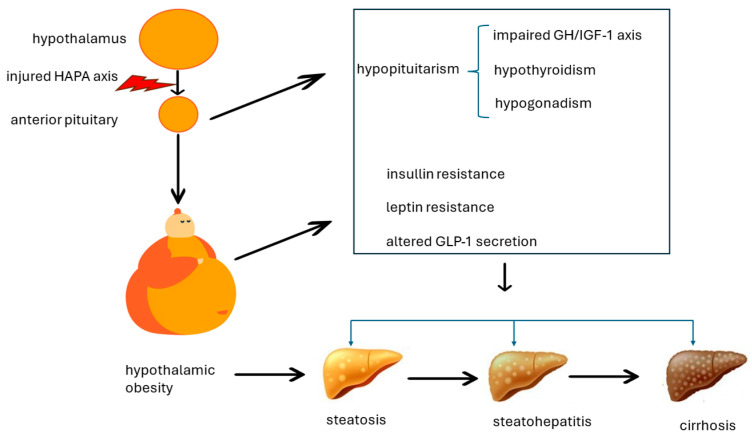
The multi-factorial pathogenesis of metabolic dysfunction-associated steatotic liver disease developing in the context of hypothalamic obesity. The pathogenesis of MASLD, MASH, and MASH-cirrhosis in the context of hypothalamic obesity reflects various forms of what was previously referred to as “Endocrine NAFLD and NASH” [8,9]. Patients with hypopituitarism experience impaired GH/IGF axis, hypogonadotropic hypogonadism, and hypothyroidism. Each of these hormonal deficits separately impacts MASLD and contributes to worsening its course [18].

The pathogenesis of MASLD developing in the context of HO is deemed to be complex and multi-factorial, as illustrated in Figure 2. 

### 4.1. Impaired Growth Hormone/Insulin-like Growth Factor-1 Axis

Released in a pulsatile fashion by the anterior pituitary, growth hormone (GH) exerts beneficial antisteatotic and antifibrotic downstream effects through decreased visceral fat and improved insulin sensitivity. This is primarily achieved through the production of insulin-like growth factor-1 (IGF-1) in hepatocytes, mediated by JAK2 and STAT5 [18]. These physiological actions mechanistically explain why an impaired GH-IGF1 axis results in the development of MASLD, both in children and adults, in the context of anterior pituitary deficiency [19,20].

Counterintuitively, a small study of 22 hypopituitary adults with GH deficiency found that GH replacement had no effect on liver fat content, as accurately assessed with proton magnetic resonance spectroscopy [21]. Analysis of data from 42 juvenile patients who underwent recombinant human GH replacement therapy after resection of craniopharyngioma showed improved metabolic parameters, high-sensitivity C reactive protein, and liver tests as surrogate biomarkers of MASLD, without providing evidence of reduced liver fat content or improved hepatic histology [22]. Therefore, the treatment of adult-onset growth hormone deficiency with hormone replacement therapy remains an individualized decision. Additional randomized, placebo-controlled studies are needed to ascertain the long-term outcomes [23].

### 4.2. Hypogonadism

Sex hormones physiologically regulate the amount and distribution of adipose tissue and govern insulin sensitivity. As a result, hypogonadism, specifically, a deficiency in sex hormones, is typically associated with complete metabolic syndrome or its individual constitutive characteristics, notably including MASLD in humans [19].

In men, physiological levels of androgens protect against MASLD by counteracting metabolic syndrome or its individual features [18]. Among the various circulating androgens, only testosterone and dihydrotestosterone (DHT) bind to the two isoforms of androgen receptors (ARs), AR-A and AR-B [24]. Androgens have both genomic and non-genomic effects. The former is achieved either through the activation of nuclear receptors, followed by binding to specific DNA motifs in its target gene, or by the androgen receptor recruiting other transcription factors such as AP-1, nuclear factor-*κ*B, sex-determining region Y, and the E26 transformation-specific family of transcription factors to bind DNA regions and participate in the transcription activation of many other genes [24]. The DNA-independent non-nuclear receptor of androgens rapidly interacts with cytoplasmic signal transduction pathways, including protein kinase A (PKA) and mitogen-activated protein kinase (MAPK)/extracellular signal-regulated protein kinase (ERK) [24]. Through these molecular mechanisms, androgens regulate the metabolism of glucose and lipids in the liver [24].

In women, physiological estradiol levels prevent insulin resistance and MASLD, which develop in ovariectomized women and are reversed by estrogen hormonal treatment [25]. Estrogen signaling is mediated by estrogen receptor (ER) isoforms ER-α and ER-β, as well as membrane-bound receptors including G protein-coupled ER (GPER, also known as GPR30) and membrane-associated ER-α and ER-β variants [26].

HO is associated with hypogonadotropic hypogonadism, leading to sexual dysfunction, infertility, and an increased risk of cardiovascular disease [27]. MASLD, secondary to hypogonadism, primarily results from increased de novo hepatic lipogenesis in both sexes [18]. However, the effects of sex hormone replacement therapy on MASLD in the context of HO are not fully understood and need further investigation.

### 4.3. Hypothyroidism

Physiologically, thyroid hormones control basal energy expenditure. This crucial role is carried out through the different tissue distribution and various biological roles of the two major thyroid receptor (TR) isoforms alpha and beta. Indirectly, they also act on a variety of other nuclear receptors, such as peroxisome proliferator-activated receptor (PPAR), liver X receptor (LXR), and bile acid signaling pathways [28].

In hepatocytes, thyroid hormones regulate energy and metabolic homeostasis by influencing all steps and pathways involved in the development of MASLD. These include the export and β-oxidation of lipids, de novo lipogenesis, hepatic insulin sensitivity, gluconeogenesis, and the metabolism of bile acids [28].

Based on these grounds, it has been proposed that because of its distinct pathogenesis, MASLD associated with hypothyroidism should be considered a specific form of NAFLD [28]. However, this proposal has yet to be included in the context of the novel MASLD nomenclature, which only considers HCV, malnutrition, celiac disease, and human immunodeficiency virus and does not address any forms of endocrine MASLD [15].

Thyroid hormone receptor-β is the main receptor isoform found in the liver and cardiac ventricles. Promising data on the efficacy of resmetirom, a selective agonist of thyroid hormone receptor-β, provides additional (though indirect) evidence of the role of the thyroid hormone in the development and progression of MASLD [29]. Therefore, future research should explore the potential benefits of resmetirom in treating MASLD associated with HO.

### 4.4. Leptin Resistance and Insulin Resistance

Leptin, a word of Greek origin meaning “lean”, is a hormone discovered in 1994 by Friedman [30]. It is a small protein hormone encoded by the *ob* gene. Individuals with obesity often exhibit hyperleptinemia, indicating leptin resistance. This resistance is more pronounced in children with HO compared with those with simple obesity [31]. Leptin resistance also correlates with more severe insulin resistance in children with HO than in those with simple obesity [31]. These findings suggest that HO is linked to dysregulated afferent (leptin) and efferent (insulin) neural outputs through the autonomic nervous system, leading to fat accumulation due to an excess of energy. A significant body of evidence has identified persistent hyperleptinemia as a pathogenic mechanism in the development of the MASLD spectrum [32].

### 4.5. Altered GLP-1 Secretion

The 30-amino acid peptide incretin glucagon-like peptide 1 (GLP-1) is synthesized by the intestinal epithelial endocrine L-cells. It is rapidly released and degraded to stimulate insulin secretion in a pulsatile manner and to inhibit glucagon secretion [33]. Thanks to these kinetic characteristics, GLP-1 contributes to limiting postprandial glucose excursions, inhibiting motor and secretory activity of the gastrointestinal tract (thus acting as part of the “ileal brake” mechanism), and acting as a physiological regulator of appetite and food intake [33]. Therefore, suboptimal GLP-1 secretion, in the context of resistance to GLP-1, is also involved in the pathogenesis of “diabesity”, and GLP-1 receptor agonists are fundamental options for the treatment of T2D and MASD [34,35]. A small case series of children with HO, compared with children with simple obesity, found that fasting and 120 min post-glucose load GLP-1 levels were significantly more elevated among children with HO. They also had significantly higher fasting triglyceridemia and higher scores of hyperphagia than controls with simple obesity [36]. These data indicate that impaired satiety plays a major role in HO and metabolic dysfunction, as reported by Brobeck several decades ago [3].

## 5. Progress in Metabolomics

Recent progress in metabolomics has contributed to promoting our understanding of the detailed pathomechanisms that ultimately lead to liver cirrhosis during HO, via MASLD, in both animal models and human studies (Table 2) [37,38,39].

The findings indicate that specific profiles are associated with MASLD that occurs in the context of hypothalamic obesity. Moreover, these metabolomics investigations have shown that hypopituitarism and hypophysectomy are associated with liver steatosis, the formation of oxidative stress, abnormal fatty oxidation, and significant changes in several lipidic compounds. These findings may be used to discriminate postoperative obesity and overweight in patients with craniopharyngioma (Table 3).

Phosphatidylethanolamine, because of its chemical structure, has a conical shape that is crucial for shaping the curvature of the inner layer of cellular and organellar membranes. It is the most abundant type of phospholipid in these membranes, helping to maintain a high degree of membrane curvature. This is essential for the efficient functioning of inner mitochondrial cristae, which in turn helps to preserve energy homeostasis and prevent insulin resistance [40].

The enzyme cytidine triphosphate:phosphoethanolamine cytidylyltransferase (Pcyt2) catalyzes the rate-limiting step in the synthesis of phosphatidylethanolamine along the Kennedy pathway. This pathway also involves diacylglycerol as a substrate in the final step of the PE-Kennedy pathway. Impaired Pcyt2 activity leads to the accumulation of diacylglycerol, increasing the risk of obesity and insulin resistance [40]. Interestingly, mice heterozygous for Pcyt2 develop MASH [41].

In their turn, ceramides and diacylglycerol play a key role in the pathogenesis of MASLD experimentally induced in mice subjected to high-fat diets [42]. Consistently, impaired catabolism of diacylglycerol and lysophosphatidylcholine in genetically engineered mice exhibiting the deletion of carboxylesterase 2a provokes hepatic steatosis and insulin resistance [43]. Collectively, these studies confirm the notion that diacylglycerol and lysophosphatidylcholine are lipotoxic molecules that accumulate in lipid droplets [44].

The first report of an independent association between MASLD and elevated serum uric acid (SUA) values dates back to 2002 [45] and has more recently been confirmed by a meta-analytic review of 50 studies involving 2,079,710 participants of whom 719,013 had MASLD [46]. Importantly, SUA levels, which are independent predictors of MASH liver histology [47], have been proposed as the sixth cardiometabolic criterion for MASLD diagnosis [48]. They, together with other variables, contribute to predicting incident MASLD outcome at 5 years [49]. Consistently, the reduction in SUA levels with allopurinol was associated with a significant decrease in the controlled attenuation parameter score, an imaging-based indicator of steatosis. This was observed in a clinical non-randomized trial involving 90 individuals with MASLD who were assigned to one of three groups for three months as follows: allopurinol 100 mg, febuxostat 40 mg daily, along with lifestyle modifications for those with high SUA levels, or lifestyle modifications alone for patients with normal SUA values [50].

**Figure 3:**
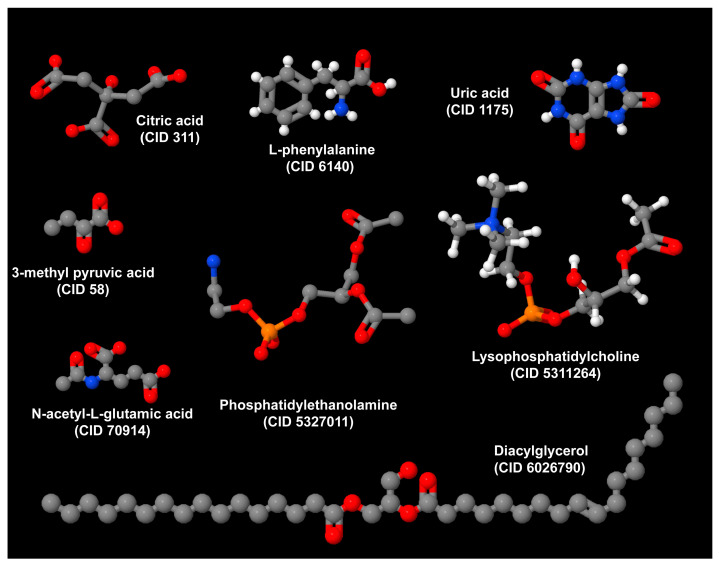
Metabolites associated with the initiation and progression of MASLD. Representative compounds of varying sizes and chemical properties are considered drivers of MASLD, indicating that the pathogenesis of MASLD is a complex molecular process. The structures shown were generated using Jmol (version 14.2.15_2015.07.09) and chemical depiction information sourced from entries stored in the PubChem Compound Database under the corresponding CID numbers [54].

L-phenylalanine has previously been implicated in the mechanism of metabolic response to hepatectomy and is a precursor in the synthesis of thyroid hormones in non-diabetic and non-obese MASLD [51,52,53]. Additional studies are necessary to characterize the mechanisms and significance of other metabolites such as citric acid, N-acetyl-L-glutamic acid, and 3-methyl pyruvic acid, in the development and progression of MASLD. Importantly, all these metabolites are highly divergent, have different sizes and belong to different classes of biomolecules (Figure 3).

## 6. Conclusions and Future Directions

Over the decades, HO has gained increasing interdisciplinary interest and has stimulated translational research. It must be considered an extraordinary experimental and clinical toolbox for exploring the connections linking neurohormonal physiology, endocrinology, metabolism, and clinical hepatology. These combinations strongly tie the history with the future of medicine. A consistent body of recent investigations has clearly contributed to defining that energy surplus, metabolic dysfunction, and lipotoxicity account for the historical observations of experimental injuries of the HAPA, culminating in HO and liver cirrhosis. This clarified paradigm recapitulates the intimate association between endocrine derangements and dysmetabolism with progressive steatotic liver disease (SLD). It is noteworthy that MASLD associated with HO, although a distinct form of SLD, has not been included under the categorization “specific etiology SLD” [15], which suggests a further refinement of this classification.

Of additional interest is gaining a better understanding of these MASLD forms occurring in the context of HO, promising to reveal novel clinical associations and innovative therapeutic approaches. These findings may potentially be utilized in selected cases of primary MASLD as well [19]. Therefore, future studies should aim to define which of the above hormonal and metabolic axes are specifically involved in each individual patient. This will open opportunities for precision medicine approaches in the field of metabolic disorders [55] through the identification of tailored hormonal replacement and innovative drug targets.

## Figures and Tables

**Table 1:** Etiology of hypothalamic obesity *.

Genetic	Prader–Willy Syndrome
Intracranial neoplasm	Craniopharingyoma, pituitary macroadenoma, glioma, meningioma, teratoma, germ cell tumor, chordoma, hystiocytosis X, hamartoma, metastasis
Iatrogenic	Surgery, radiation therapy, subthalamic implants
Trauma	Head trauma
Inflammatory conditions	Sarcodosis, arachnoditis, encephalitis, tuberculosis
Vascular	Aneurysm of the internal carotid artery
Others	Hypodipsia–hypernatremia syndrome

* data were taken from [11,12].

**Table 2:** Recent metabolomics investigations addressing hepatic involvement associated with hypothalamic obesity.

Species	Findings
Rats	In rats, there is an unbalanced ratio of palmitoyl acid, dehydrocholic acid, and 7-ketolithocholic acid, all of which are increased, while lysophosphatidylcholine and fatty acid esters of hydroxy fatty acids are decreased [37]. Additionally, mitochondrial dysfunction, oxidative stress, lipid peroxidation, and reduced glutathione are all increased [38].
Humans	In humans, phosphatidylethanolamine (16:0e/22:4), ceramides [(d16:0/16:0) and (d18:1/16:0)], and diacylglycerol (18:2/20:4) predict HO among patients with craniopharyngioma treated with surgery [39]. Additional predictors include citric acid, serum uric acid, *N*-acetylglutamic acid, 3-methyl pyruvic acid, and L-phenylalanine [39]. Phosphatidylethanolamine, ceramides, and diacylglycerol play a key role in the pathogenesis of MASLD.

**Table 3:** Alterations observed in hypopituitarism or after hypophysectomy.

Method	Findings	Comment	Reference
Ten Lewis dwarf homozygous (dw/dw) rats, and ten Lewis dwarf heterozygous (dw/+) rats were analyzed.	dw/dw rats exhibited more pronounced hepatic steatosis accompanied by higher serum transaminase values. Among dw/dw rats, compared with dw/+ rats, levels of LPC 16:2, LPC 18:3, LPC 22:6, and FAHFA18:1 were significantly decreased, while levels of palmitoyl acid, dehydrocholic acid, and 7-ketolithocholic acid were significantly increased	Distinctive hepatic metabolic profiles are associated with liver steatosis and elevated transaminases in Lewis dw/dw rats with congenital IGHD.	[37]
Serum untargeted metabolomics was evaluated in male rats in which HP was induced by hypophysectomy, followed by rhGH HRT replacement therapy.	Among rats with HP, biomarkers of mitochondrial dysfunction and oxidative stress were significantly increased compared with age-matched controls. Additionally, hypophysectomy was associated with severe hepatic steatosis, lipid peroxidation, and reduced levels of GSH, which were subsequently modulated by rhGH HRT. Proteomic analysis identified cytochrome P450s, mitochondrial translation elongation, and PPARA-activating genes as the major distinguishing pathways in hypophysectomized rats. Downregulation of JAK2-STAT5B and upregulation of mTOR signaling pathway.	This study demonstrates an imbalance in oxidative stress resulting from abnormal fatty acid oxidation and NADPH regeneration in a rat model of MASLD associated with hypophysectomy.	[38]
Serum metabolomics and lipidomics were compared across three BMI categories in 120 postoperative Chinese adult patients with CP.	CA and SUA had predictive potential for postoperative obesity and overweight in patients with CP, while N-acetylglutamic acid, 3-methyl pyruvic acid, and L-phe precisely predicted the occurrence of postoperative obesity in patients with CP. PE (16:0e/22:4), Cer (d16:0/16:0), DG (36:2e), Cer (d18:1/16:0), and DG (18:2/20:4) were identified as potential predictors for postoperative obesity in patients with CP.	In patients with CP, the leading cause of HO, metabolomics and lipidomics offer a promising tool for discriminating the occurrence of postoperative obesity, with lipidomics exhibiting higher sensitivity.	[39]

List of abbreviation used: BMI, body mass index; CA, citric acid; Cer, ceramides; CP, craniopharyngioma; DG, diacylglycerol; dw, dwarf; FAHFA, fatty acid esters of hydroxy fatty acids; GSH, reduced glutathione; HO, hypothalamic obesity; HP, hypopituitarism; HRT, hormonal replacement therapy IGHD, isolated growth hormone deficiency; LPC, lysophosphatidylcholine; SUA, serum uric acid; PE, phosphatidylethanolamine; phe, phenylalanine; rhGH, recombinant human growth hormone.

## Abbreviations

AR(s)	androgen receptor(s)
FFA(s)	free fatty acid(s)
GH	growth hormone
GLP-1	glucagon-like peptide 1
IGF-1	insulin-like growth factor-1
HAPA	hypothalamic-anterior pituitary axis
HO	hypothalamic obesity
MASLD	metabolic dysfunction-associated steatotic liver disease
MASH	metabolic dysfunction-associated steatohepatitis
Pcyt2	cytidine triphosphate:phosphoethanolamine cytidylyltransferase
SUA	uric acid
T2D	type 2 diabetes

## References

[1] Rusch J.A., Layden B.T., Dugas L.R. (2023). Signalling cognition: The gut microbiota and hypothalamic pituitary-adrenal axis. Front. Endocrinol..

[2] Wasilewska N., Lebensztejn D.M. (2021). Non-alcoholic fatty liver disease and lipotoxicity. Clin. Exp. Hepatol..

[3] Brobeck J.R. (1946). Mechanism of the development of obesity in animals with hypothalamic lesions. Physiol. Rev..

[4] Lee Y., Hirose H., Ohneda M., Johnson J.H., McGarry J.D., Unger R.H. (1994). Beta-cell lipotoxicity in the pathogenesis of non-insulin-dependent diabetes mellitus of obese rats: Impairment in adipocyte beta-cell relationships. Proc. Natl. Acad. Sci. USA.

[5] Weinberg J.M. (2006). Lipotoxicity. Kidney Int..

[6] Sørensen T.I., Virtue S., Vidal-Puig A. (2010). Obesity as a clinical and public health problem: Is there a need for a new definition based on lipotoxicity effects?. Biochim. Biophys. Acta.

[7] Adams L.A., Feldstein A., Lindor K.D., Angulo P. (2004). Nonalcoholic fatty liver disease among patients with hypothalamic and pituitary dysfunction. Hepatology.

[8] Lonardo A., Carani C., Carulli N., Loria P. (2006). ‘Endocrine NAFLD’ a hormonocentric perspective of nonalcoholic fatty liver disease pathogenesis. J. Hepatol..

[9] Loria P., Carulli L., Bertolotti M., Lonardo A. (2009). Endocrine and liver interaction: The role of endocrine pathways in NASH. Nat. Rev. Gastroenterol. Hepatol..

[10] Lee M., Korner J. (2009). Review of physiology, clinical manifestations, and management of hypothalamic obesity in humans. Pituitary.

[11] Roth C.L., McCormack S.E. (2024). Acquired hypothalamic obesity: A clinical overview and update. Diabetes Obes. Metab..

[12] Hochberg I., Hochberg Z. (2010). Hypothalamic obesity. Endocr. Dev..

[13] Romigi A., Feola T., Cappellano S., De Angelis M., Pio G., Caccamo M., Testa F., Vitrani G., Centonze D., Colonnese C. (2022). Sleep Disorders in Patients With Craniopharyngioma: A Physiopathological and Practical Update. Front. Neurol..

[14] Lonardo A., Leoni S., Alswat K.A., Fouad Y. (2020). History of Nonalcoholic Fatty Liver Disease. Int. J. Mol. Sci..

[15] Rinella M.E., Lazarus J.V., Ratziu V., Francque S.M., Sanyal A.J., Kanwal F., Romero D., Abdelmalek M.F., Anstee Q.M., Arab J.P. (2023). A multisociety Delphi consensus statement on new fatty liver disease nomenclature. Hepatology.

[16] Malhi H., Gores G.J. (2008). Molecular mechanisms of lipotoxicity in nonalcoholic fatty liver disease. Semin. Liver Dis..

[17] Tilg H., Adolph T.E., Moschen A.R. (2021). Multiple parallel hits hypothesis in nonalcoholic fatty liver disease: Revisited after a decade. Hepatology.

[18] Hutchison A.L., Tavaglione F., Romeo S., Charlton M. (2023). Endocrine aspects of metabolic dysfunction associated steatotic liver disease (MASLD): Beyond insulin resistance. J. Hepatol..

[19] Lonardo A., Mantovani A., Lugari S., Targher G. (2019). NAFLD in some common endocrine diseases: Prevalence, pathophysiology, and principles of diagnosis and management. Int. J. Mol. Sci..

[20] Liebe R., Esposito I., Bock H.H., Vom Dahl S., Stindt J., Baumann U., Luedde T., Keitel V. (2021). Diagnosis and management of secondary causes of steatohepatitis. J. Hepatol..

[21] Meienberg F., Yee M., Johnston D., Cox J., Robinson S., Bell J.D., Thomas E.L., Taylor-Robinson S.D., Godsland I. (2016). Liver fat in adults with GH deficiency: Comparison to matched controls and the effect of GH replacement. Clin. Endocrinol..

[22] Li S., Wang X., Zhao Y., Nie M., Ji W., Mao J., Wu X. (2022). Metabolic effects of recombinant human growth hormone replacement therapy on juvenile patients after craniopharyngioma tesection. Int. J. Endocrinol..

[23] He X., Barkan A.L. (2020). Growth hormone therapy in adults with growth hormone deficiency: A critical assessment of the literature. Pituitary.

[24] Shen M., Shi H. (2015). Sex hormones and their receptors regulate liver energy homeostasis. Int. J. Endocrinol..

[25] Zhu L., Brown W.C., Cai Q., Krust A., Chambon P., McGuinness O.P., Stafford J.M. (2013). Estrogen treatment after ovariectomy protects against fatty liver and may improve pathway-selective insulin resistance. Diabetes.

[26] Fuentes N., Silveyra P. (2019). Estrogen receptor signaling mechanisms. Adv. Protein Chem. Struct. Biol..

[27] Zhou Z., Zhang S., Hu F. (2021). Endocrine disorder in patients with craniopharyngioma. Front. Neurol..

[28] Lonardo A., Ballestri S., Mantovani A., Nascimbeni F., Lugari S., Targher G. (2019). Pathogenesis of hypothyroidism-induced NAFLD: Evidence for a distinct disease entity?. Dig. Liver Dis..

[29] Harrison S.A., Bedossa P., Guy C.D., Schattenberg J.M., Loomba R., Taub R., Labriola D., Moussa S.E., Neff G.W., Rinella M.E. (2024). A Phase 3, randomized, controlled trial of Resmetirom in NASH with liver fibrosis. N. Engl. J. Med..

[30] Li M.D. (2011). Leptin and beyond: An odyssey to the central control of body weight. Yale J. Biol. Med..

[31] Guran T., Turan S., Bereket A., Akcay T., Unluguzel G., Bas F., Gunoz H., Saka N., Bundak R., Darendeliler F. (2009). The role of leptin, soluble leptin receptor, resistin, and insulin secretory dynamics in the pathogenesis of hypothalamic obesity in children. Eur. J. Pediatr..

[32] Jiménez-Cortegana C., García-Galey A., Tami M., Del Pino P., Carmona I., López S., Alba G., Sánchez-Margalet V. (2021). Role of leptin in non-alcoholic fatty liver disease. Biomedicines.

[33] Holst J.J. (2007). The physiology of glucagon-like peptide 1. Physiol. Rev..

[34] McGlone E.R., Bloom S.R., Tan T.M. (2024). Glucagon resistance and metabolic-associated steatotic liver disease: A review of the evidence. J. Endocrinol..

[35] Nevola R., Epifani R., Imbriani S., Tortorella G., Aprea C., Galiero R., Rinaldi L., Marfella R., Sasso F.C. (2023). GLP-1 receptor agonists in non-alcoholic fatty liver disease: Current evidence and future perspectives. Int. J. Mol. Sci..

[36] Chai-Udom R., Aroonparkmongkol S., Sahakitrungruang T. (2020). Metabolic features and changes in glucose-induced serum glucagon-like peptide-1 levels in children with hypothalamic obesity. J. Pediatr. Endocrinol. Metab..

[37] Guo X., Hu W., Lyu X., Xu H., Zhu H., Pan H., Wang L., Yang H., Gong F. (2024). The distinct hepatic metabolic profile and relation with impaired liver function in congenital isolated growth hormone-deficient rats. Endocr. Connect..

[38] Zhang Y., Chen P., Fang X. (2024). Proteomic and metabolomic analysis of GH deficiency-induced NAFLD in hypopituitarism: Insights into oxidative stress. Front. Endocrinol..

[39] Zhang Q., Feng Y., Wu D., Xie Y., Wu G., Wu W., Wang H., Liu X., Fan L., Xiang B. (2024). Serum metabolomic and lipidomic profiling reveals the signature for postoperative obesity among adult-onset craniopharyngioma. Metabolites.

[40] Duncan R.E. (2023). Deficiency of phosphatidylethanolamine synthesis: Consequences for skeletal muscle. Function.

[41] Fullerton M.D., Hakimuddin F., Bonen A., Bakovic M. (2009). The development of a metabolic disease phenotype in CTP:phosphoethanolamine cytidylyltransferase-deficient mice. J. Biol. Chem..

[42] Mourad S., Abdualkader A.M., Li X., Jani S., Ceddia R.B., Al Batran R. (2024). A high-fat diet supplemented with medium-chain triglycerides ameliorates hepatic steatosis by reducing ceramide and diacylglycerol accumulation in mice. Exp. Physiol..

[43] Chalhoub G., Jamnik A., Pajed L., Kolleritsch S., Hois V., Bagaric A., Prem D., Tilp A., Kolb D., Wolinski H. (2023). Carboxylesterase 2a deletion provokes hepatic steatosis and insulin resistance in mice involving impaired diacylglycerol and lysophosphatidylcholine catabolism. Mol. Metab..

[44] Preuss C., Jelenik T., Bódis K., Müssig K., Burkart V., Szendroedi J., Roden M., Markgraf D.F. (2019). A new targeted lipidomics approach reveals lipid droplets in liver, muscle and heart as a repository for diacylglycerol and ceramide species in non-alcoholic fatty liver. Cells.

[45] Lonardo A., Loria P., Leonardi F., Borsatti A., Neri P., Pulvirenti M., Verrone A.M., Bagni A., Bertolotti M., Ganazzi D. (2002). Policentrica Steatosi Epatica Non Alcolica. Fasting insulin and uric acid levels but not indices of iron metabolism are independent predictors of non-alcoholic fatty liver disease. A case-control study. Dig. Liver Dis..

[46] Sun Q., Zhang T., Manji L., Liu Y., Chang Q., Zhao Y., Ding Y., Xia Y. (2023). Association between serum uric acid and non-alcoholic fatty liver disease: An updated systematic review and meta-analysis. Clin. Epidemiol..

[47] Ballestri S., Nascimbeni F., Romagnoli D., Lonardo A. (2016). The independent predictors of non-alcoholic steatohepatitis and its individual histological features.: Insulin resistance, serum uric acid, metabolic syndrome, alanine aminotransferase and serum total cholesterol are a clue to pathogenesis and candidate targets for treatment. Hepatol. Res..

[48] He L., Qiu K., Zheng W., Kong W., Zeng T. (2024). Uric acid may serve as the sixth cardiometabolic criterion for defining MASLD. J. Hepatol..

[49] Gao L., Cui W., Mu D., Li S., Li N., Zhou W., Hu Y. (2024). Nomogram for predicting 5-year metabolic dysfunction-associated steatotic liver disease risk: Retrospective cohort study. Endocr. Connect..

[50] Al-Shargi A., El Kholy A.A., Adel A., Hassany M., Shaheen S.M. (2023). Allopurinol versus Febuxostat: A new approach for the management of hepatic steatosis in metabolic dysfunction-associated steatotic liver disease. Biomedicines.

[51] He R., Gao S., Yao H., Zhao Z., Tong J., Zhang H. (2023). Mechanism of metabolic response to hepatectomy by integrated analysis of gut microbiota, metabolomics, and proteomics. Microbiol. Spectr..

[52] Chen Y., Li C., Liu L., Guo F., Li S., Huang L., Sun C., Feng R. (2016). Serum metabolomics of NAFLD plus T2DM based on liquid chromatography-mass spectrometry. Clin. Biochem..

[53] Demirel M., Köktaşoğlu F., Özkan E., Dulun Ağaç H., Gül A.Z., Sharifov R., Sarıkaya U., Başaranoğlu M., Selek Ş. (2023). Mass spectrometry-based untargeted metabolomics study of non-obese individuals with non-alcoholic fatty liver disease. Scand. J. Gastroenterol..

[54] National Library of Medicine PubChem Compound. https://www.ncbi.nlm.nih.gov/pccompound/.

[55] Lonardo A., Byrne C.D., Targher G. (2021). Precision medicine approaches in metabolic disorders and target organ damage: Where are we now, and where are we going?. Metab. Target. Organ. Damage.

